# Segmentation of ovarian cancer tumors from CT images based on a two-stage neural network fusion system

**DOI:** 10.3389/frai.2026.1974931

**Published:** 2026-10-14

**Authors:** Miruna-Cristina Andone, Loretta Ichim, Dan Popescu

**Affiliations:** 1 Faculty of Automatic Control and Computers, National University of Science and Technology Politehnica of Bucharest, Bucharest, Romania; 2 Stefan S. Nicolau Institute of Virology, Romanian Academy, Bucharest, Romania

**Keywords:** computed tomography, ensemble strategies, fusion decision, neural networks, ovarian cancer, tumor segmentation

## Abstract

The detection of ovarian cancer using computed tomography images is making rapid progress, combining the strengths of different neural networks within a complex ensemble, a more effective model can be created to support medical decision-making in ovarian cancer cases. Various neural networks and ensemble models were tested on the TCGA OV database. Tumor regions were segmented using computer-assisted annotation masks, while molecular subtype labels were assigned at the patient level based on TCGA genomic classifications and mapped to tumors. As the main novelty, the proposed method involves a two-stage fusion system. The first stage combines several neural networks into four ensembles to capture diverse features, and the second stage integrates these results into a global system to enhance segmentation accuracy. The selected models demonstrated strong segmentation performance within the investigated experimental setting and were subsequently integrated into the proposed two-stage fusion framework. The neural networks for the first stage are Attention U-Net, DeepLabV3+, and ViT. The results show that the proposed two-stage fusion system achieved higher observed segmentation performance than the evaluated individual neural networks and first-stage ensemble configurations within the present TCGA-OV experimental setting. Across the five evaluated classes, the Global System achieved an average F1 score of 93.53%, recall of 93.38%, and precision of 93.68%, supporting the technical feasibility of the proposed two-stage fusion approach for ovarian tumor segmentation within the investigated dataset.

## 1 Introduction

According to epidemiological data from GloboCan-Global Cancer Observatory, ovarian cancer (OC) is the eighth most common cancer in women and the 18th most common cancer in the general population. It accounts for approximately 4.7% of all cancer deaths in women and is the deadliest gynecological cancer, according to the World Health Organization. Epithelial ovarian cancer (Lheureux et al., 2019) is the most common type of OC (95% of ovarian malignancies). Long-term survival rates remain low even in the context of advances in cancer therapies (International Agency for Research on Cancer, 2024). There are many risk factors, such as age, specific inherited genotype characteristics family history, obesity, hormone therapy during menopause, endometriosis, etc. (American Cancer Society, 2020).

Ovarian cancer is rarely identified in early stages. Histopathological analysis is the most precise and accurate method (Saha et al., 2025) to detect these OC stages. Due to the lack of symptoms, patients are usually presented in late stages, when surgery can no longer be performed, and the treatment of metastatic patients is ineffective or almost impossible. Approximately 150,000 women die annually globally. Thus, patients present to the hospital with symptoms characteristic of the terminal stages, although assessments that enable early diagnosis are available and important (World Ovarian Cancer Coalition, 2024). The latest published statistics show that in 2022, 324,603 women were diagnosed with OC, and predictions made by the World Ovarian Cancer Coalition based on data provided by Globocan show that four million lives will be lost by 2040, and by 2050, approximately 12 million new cases will be diagnosed. Therefore, OC has been declared a global health priority by specialist organizations. Early detection can reduce the costs of medical treatment and increase its efficiency through screening programs, augmented by artificial intelligence (AI) algorithms. Even though notable progress has been made, the reported 5-year survival rate, indicative of OC, is below 30% for diagnosis in late stages (Wu et al., 2023).

Traditional methods of diagnosing and staging the disease, which rely on manual imaging analysis, have limitations due to subjectivity and the risk of human error, are expensive (Wang et al., 2024), and cannot be applied on a large scale for screening processes (Boyanapalli and Shanthini, 2023). OC detection and classification from CT scans by combining AI techniques is gaining momentum. Several comparative studies are being conducted between the performance of software systems (Maslej et al., 2024), physician interpretations, and the performance of physician interpretations augmented by AI (Ma et al., 2023). The development of deep learning algorithms for automated image analysis has offered new opportunities, including for early and accurate OC detection (Sundar et al., 2024). In recent years, spectacular progress has been made in the design of high-performance neural networks (NNs) for medical imaging (Bharadwaj et al., 2024). Efforts are underway, particularly in the early stages, to utilize public databases and develop synthetic data augmentation methods, such as Generative Adversarial Networks (GANs).

There are different classifications to characterize and stage ovarian tumors to plan management and treatment. The European Society of Urogenital Radiology (ESUR) guidelines state that the FIGO (Fédération Internationale de Gynécologie et d'Obstétrique) staging system based on CT scans is combined with specific annotations that divide tumors into malignant subclasses such as differentiated, proliferative, immunoreactive, and mesenchymal. They are primarily defined through molecular and transcriptomic profiling. With the possibility of calculating a predictability score, for example, based on the ROMA algorithm (Risk of Ovarian Malignancy Algorithm) (Bhuvaneshwari et al., 2024), the data can be integrated to identify (early) OC. Studies have been conducted to improve early diagnosis and test accuracy; for example, the ROCkeTS (Refining Ovarian Cancer Test accuracy Scores) study (Sundar et al., 2016).

Although AI cannot replace medical decision-making, it can create models with an impact on economic and social levels (Jan et al., 2023). Bharadwaj et al. (2024) reported a 5-year return on investment of 451% for hospital-based artificial intelligence implementation. AI applied in medicine has a great impact on radiology. Depending on the images available, the specialist can quickly identify tumor structures of various sizes, including small ones. Introducing multidimensional information through the analysis of radiomic parameters (Naderi Yaghouti et al., 2025) can extend beyond the classic interpretation of tumors by radiologists (Minaee et al., 2022).

Therefore, clinical examinations must be accompanied by imaging explorations, such as CT and MRI examinations, which guide the diagnosis of detected tumor masses, along with morphopathological explorations, thus leading to a rapid and accurate diagnosis made in the initial stages (Holback et al., 2016). CT examinations are considered standard for the diagnosis, staging, and follow-up of ovarian tumors. In a clinical context, data provided by AI algorithms can intervene in the OC diagnosis of patients through social and economic impact. By testing ensemble algorithms created by combining multiple models, the new trend proposes generating more robust and accurate predictions by reducing the errors of individual models and increasing stability on varied data, including incomplete or noisy data (Arathi and Shanthini, 2022). Another advantage is the flexibility to develop algorithms for various data, enabling the integrated implementation of multiple datasets (Zhou et al., 2020).

This paper addresses ovarian tumor segmentation from CT images using a two-level neural network fusion framework. Importantly, the five-class formulation used in this study should not be interpreted as direct voxel-wise prediction of molecular subtypes from CT images. The spatial tumor mask represents the anatomical tumor region, whereas the Differentiated, Proliferative, Mesenchymal, and Immunoreactive labels originate from patient-level TCGA molecular classifications and were subsequently assigned to the corresponding tumor masks. Thus, the proposed task combines anatomical tumor segmentation with patient-level subtype-derived labeling for multi-class analysis. The study therefore evaluates how accurately the models reproduce these mapped labels within the tumor regions, rather than claiming that CT images independently resolve transcriptomic heterogeneity at the voxel level. As a novelty, it presents solutions for ovarian tumor segmentation through a two-stage fusion system. The principal performance indicators used for comparative evaluation were precision, recall, and F1/Dice score. These metrics were used consistently for network ranking, ensemble weighting, and comparison across the successive fusion stages. Accuracy and Intersection over Union (IoU) were considered complementary segmentation measures where available. Accordingly, the main comparative results focus on F1, recall, and precision, which constitute the common evaluation criteria across the individual networks and ensemble configurations. The best NNs were selected to implement the system. To train the algorithms, annotated medical image databases containing OC cases are required. For confidentiality and ethical reasons, the legislation regarding the protection of personal data must be respected. The NN ensemble offers increased robustness by compensating for the deficiencies of the individual models. The performance of the ensemble is evaluated based on the same indicators used for the individual models, thus demonstrating the improvements made. In addition to the introduction, the article includes five other sections. Section 2 refers to the related work in the field. Section 3 presents materials and methods, such as the database and NNs used. Next, the proposed two-stage fusion system is detailed. Section 4 includes the experimental results obtained. Based on these results, Section 5 presents the discussion. Section 6 presents the conclusions.

## 2 Related works

As a support system, deep NNs can help automate and improve the diagnostic process. The ViT and Attention U-Net used for detection and automatic segmentation of ovarian tumors in CT images achieved a sensitivity of 68%, a specificity of 89%, and a diagnostic accuracy of 82%, which can aid in medical decision-making. These have even been compared to the medical performance of doctors with less than 2 years of experience in radiology, who showed a sensitivity of 67%, a specificity of 65%, and an accuracy of 66% on the same set of images (Jan et al., 2023). Some studies have reported accuracy rates of up to 95.7% by combining different NNs (Kodipalli et al., 2023). For early identification, detection, staging, and characterization of OC, non-invasive imaging methods such as CT, ultrasound, and magnetic resonance imaging (MRI) scans are largely used. A review of methods for detecting OC from such images using AI over the last 10 years is presented in (Naderi Yaghouti et al., 2025).

The use of NNs has led to advances in the detection of OC by analyzing and interpreting computed tomography images, as well as in the detection of ovarian tumors. Combining different NNs, such as ResNet50 and MobileNetV3 (Wang et al., 2023), for OC identification resulted in an improvement in accuracy of over 85%. The accuracy of malignant tumor detection increased for the integrated ensemble models, compared to single models. A DSSGL-DCNN architecture (Deep Semi-Supervised Generative Learning–Deep Convolutional Neural Network) that integrates GAN and CNN to increase the performance of the algorithms was used. Studies conducted on public data from the TCGA-OV (The Cancer Genome Atlas – ovarian cancer collection) archive utilized the native database (Holback et al., 2016), while also expanding the training datasets using data augmentation techniques.

Beyond ovarian cancer imaging, ensemble and comparative deep-learning strategies have also been investigated across different medical-image analysis tasks. Butt et al. proposed an ensemble deep-learning approach for breast cancer detection in ultrasound imaging, demonstrating the potential of combining complementary neural-network architectures to improve computer-assisted image analysis (Butt et al., 2024). Comparative approaches have also been investigated for skin lesion analysis, where machine-learning and deep-learning architectures were evaluated within a common diagnostic framework, highlighting the relevance of CNN-based representations for medical-image classification (Khan et al., 2021). Deep-learning methods have further been applied to automated skin cancer classification, supporting the broader applicability of convolutional architectures in computer-assisted diagnosis (Ashraf et al., 2020). In neurological imaging, multi-scale deep-learning strategies have been investigated for the early detection of Alzheimer's disease using functional MRI, illustrating the potential benefit of extracting complementary information at different representation scales (Butt et al., 2024). Although these studies address different diseases and imaging modalities, they provide complementary methodological context for the comparative evaluation of neural-network architectures, multi-scale feature representation, and ensemble-based decision strategies used in medical AI.

By applying protocols, constraints, and data evaluation, the generation/ augmentation of useful and high-quality data can be managed. These are intended to improve the performance and safety of OC detection algorithms. The size, location, and certain characteristics of tumors can be rapidly staged, classified, segmented, and used to predict tumor progression (Breen et al., 2023). Performance metrics such as accuracy, sensitivity, precision, F1 value, Receiver Operating Characteristic (ROC) curve, and Area Under the Curve (AUC) are used to compare different algorithms.

From a recent analysis of 28 studies, which present different NNs used in the detection of OC, the true positive prediction rates in the detection of malignant or benign case diagnosis within the framework of imaging investigations were between 80 and 92%. This demonstrates considerable performance in detecting heterogeneous benign, borderline, and malignant tumors (Piedimonte et al., 2025). Most approaches were for diagnosing malignant or benign cases (Breen et al., 2023). Obviously, being a binary classification, the results had better performance (75–99% accuracy) than those with more classes.

Several works have performed comparative analyses between different NN architectures to establish their performance. These studies are essential for determining the best NNs, as well as for developing new methods and combinations to meet clinical requirements. To this end, U-Net, ResNet, VGG, GoogLeNet, DenseNet-121, SegNet, Vision Transformer (ViT), DeepLabV3+, and Attention U-Net have been applied to various malignant pathologies, including OC (Siddique et al., 2021). To increase overall efficiency, the models can integrate the outputs of multiple architectures (Minaee et al., 2022). The authors in Kodipalli et al. (2023) made a comparative study of algorithms for ovarian tumor classification from CT images in two stages. The first stage is image segmentation using a U-Net, and the second stage is tumor classification using machine learning algorithms. However, even with the progress made in the past years in NN application for OC detection, several problems arise when the systems are used in clinical practice. The difficulties consist of the diversity of imaging data, differences in tumor characteristics, annotation, parameters analyzed, and analysis of CT images.

## 3 Materials and methods

### 3.1 Dataset used

TCGA-OV, a public database, through the medical imaging archive TCIA (The Cancer Imaging Archive), was used in this paper (Holback et al., 2016). The data consist of CT and MRI images in DICOM format, to which detailed clinical and genetic data can be correlated. The collection comprises diverse images acquired from multiple institutions using various types of scanners via different acquisition protocols (Cardoso et al., 2022). The TCGA-OV collection used in this study comprised 143 patients and 26,570 CT images. The dataset was organized for two related but conceptually distinct tasks. First, tumor segmentation was performed using computer-assisted tumor masks that define the anatomical tumor region on CT images. Second, for patients for whom a TCGA molecular classification was available, the corresponding patient-level molecular subtype label was assigned to the tumor mask. The four subtype-derived labels considered were Differentiated, Proliferative, Mesenchymal, and Immunoreactive. Therefore, these four labels should not be interpreted as independently annotated voxel-level imaging phenotypes. Rather, every pixel belonging to the tumor mask inherited the molecular subtype label of the corresponding patient, while pixels outside the tumor mask were assigned to the No Tumor class. The resulting five-class representation was consequently used as a computational formulation for evaluating tumor segmentation together with subtype-derived patient-level labeling.

Accordingly, the present framework distinguishes anatomical tumor segmentation, patient-level molecular subtype information, and propagation of the patient-level subtype label to the corresponding tumor pixels for construction of the composite five-class target representation. Examples from the TCGA-OV database used in this paper are shown in Figure 1.

**Figure 1:**
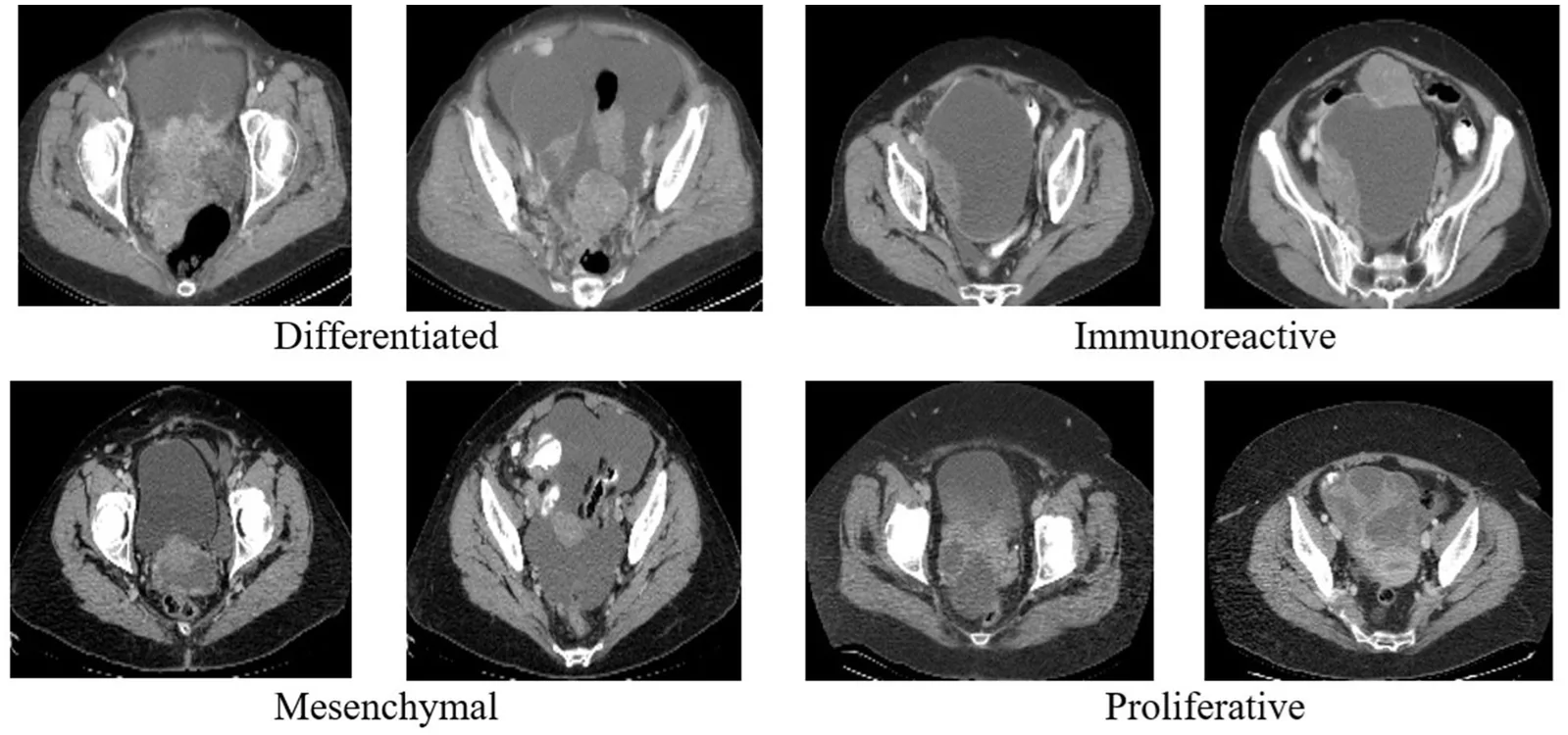
Examples of images with tumors from the TCGA-OV database.

Based on these properties, robust and generalizable models can be built for the segmentation of ovarian tumors, especially the ovarian serous carcinoma subtype. Five classes are considered: no Tumor, Differentiated, Proliferative, Mesenchymal, and Immunoreactive. These images include CT scans analyzed in a multi-institutional study. Accordingly, the No Tumor class was generated from non-tumor pixels outside the ground-truth tumor masks, whereas the four remaining classes were assigned only to pixels within tumor masks according to the patient-level TCGA molecular subtype. The mapping procedure was performed at the patient level. For each patient, the TCGA molecular subtype recorded in the genomic metadata was linked to the corresponding imaging record using the patient-level identifier. The molecular label was then associated with the tumor mask belonging to that patient. Consequently, all pixels within the verified tumor mask inherited the molecular subtype label of that patient, whereas pixels outside the tumor mask retained the No Tumor label. No separate voxel-wise molecular annotation was available, and no molecular subtype was assigned from CT appearance alone. The distribution of images used can be seen in Figure 2. To comply with confidentiality and ethical protocols, public databases such as TCGA OV are used for algorithm development. This has been annotated in advance.

**Figure 2:**
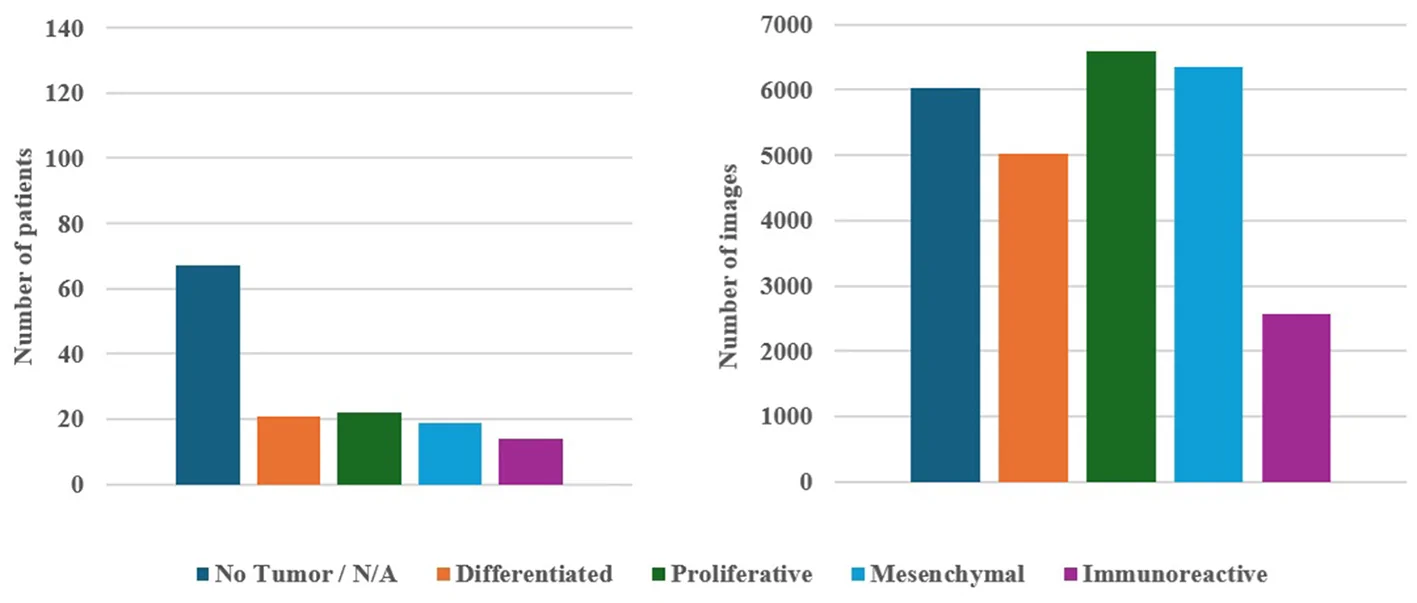
Distribution of tumor types in TCGA-OV.

The designation ‘No Tumor/N/A' used in the previous version of Figure 2 was ambiguous and has therefore been corrected. In the present five-class formulation, the No Tumor class refers exclusively to pixels outside the anatomically annotated tumor region. It does not represent patients with unavailable molecular subtype information, and missing molecular subtype information was not treated as equivalent to the absence of tumor.

All CT slices belonging to a single patient were assigned exclusively to one subset, resulting in a 70% training, 20% validation, and 10% testing split. The dataset partition was performed exclusively at the patient level. Consequently, all CT slices belonging to the same patient were assigned to a single cohort, and no patient contributed images to more than one of the training, validation, or testing subsets. This distinction is important because individual CT slices and pixels originating from the same patient are correlated observations and should not be interpreted as independent samples. All images originating from the same patient were assigned exclusively to one subset, preventing patient-level overlap between training, validation, and testing data. Nevertheless, given the total cohort of 143 patients, the independent testing subset contains a relatively limited number of subjects. Therefore, the number of image slices and pixels evaluated should not be interpreted as an equivalent number of statistically independent observations.

The tumor masks used in this study were obtained as the available ground-truth annotations associated with the imaging data and were used to define the anatomical tumor regions. The available annotations were subjected to visual verification before being used in the segmentation experiments, with the purpose of checking the anatomical plausibility and consistency of the delineated tumor regions across the CT slices. The molecular subtype information was not used to generate the anatomical tumor boundaries; instead, the patient-level TCGA molecular classification was subsequently mapped to the corresponding annotated tumor region. Thus, the anatomical ground truth and the molecular subtype information originated from two distinct annotation sources. Molecular subtype labels were subsequently mapped to tumor masks at the patient level within an annotation framework.

### 3.2 Neural network models

Several architectures that have proven effective in tumor segmentation in medical imaging were tested, such as U-Net, ResNet, VGG 16, GoogLeNet, DenseNet-121, SegNet, Vision Transformer (ViT), DeepLabV3+, and Attention U-Net.

Characterized by its “U”-shaped structure, U-Net uses an encoder, which extracts features from the input image, and a decoder, which reconstructs the segmented image. The information transfer between the corresponding levels of the encoder and decoder is achieved through skip connections (Ronneberger et al., 2015). This preserves fine details, making this architecture particularly effective in highlighting tumor contours (Siddique et al., 2021) and isolating ovarian masses (Kodipalli et al., 2023).

Attention U-Net differs from the classical U-Net architecture by introducing attention mechanisms that focus on relevant areas of the image, ignoring insignificant regions (Oktay et al., 2018). In identifying OC, Attention U-Net provides precise segmentation, focusing on tumor edges and abnormal structures to differentiate tumor tissues from healthy ones, increasing the sensitivity and specificity of detection (Wang et al., 2023).

SegNet is a specialized NN for accurate semantic segmentation of tumor masses from CT and MRI images. It uses encoding layers followed by symmetric decoding to reconstruct segmented images (Badrinarayanan et al., 2017). Its main feature is its memory efficiency, which makes this NN ideal for high-resolution DICOM medical data (Cardoso et al., 2022).

ResNet is characterized by using residual blocks to solve the gradient degradation problem (He et al., 2016). ResNet-50 is frequently used for OC detection, classification, and segmentation due to its ability to learn complex tumor representations in deep NNs (Jan et al., 2023). Residual connections within the NN facilitate stable learning (Minaee et al., 2022). The depth of this NN allows the capture of fine details—ovarian tumors are small in early stages and must be captured at a detailed level.

VGG (Visual Geometry Group) architecture is a classic deep convolutional neural network (CNN) architecture consisting mainly of stacked 3 × 3 convolution filters and 2 × 2 max-pooling layers (Simonyan and Zisserman, 2015). It is simple to understand and easy to implement due to its repetitive structure. VGG networks, such as VGG-16, are based on small 3 × 3 filters, which makes the NNs efficient in detecting small tumors. In the context of OC, VGG is predominantly used for classification and detection. Compared to ResNet or DenseNet, it has a more stable performance for different tumors (Breen et al., 2023).

The main idea of the GoogLeNet model is the use of Inception modules with different receptive fields operating in parallel, implemented efficiently through 1 × 1 convolutions (Szegedy et al., 2015). It is a 22-layer deep NN and uses two auxiliary classifiers to stabilize training.

DenseNet (Densely Connected CNNs) improves connectivity within CNN layers by connecting each layer to all previous layers in a feed-forward manner (Huang et al., 2017). This highly connected structure produces L(L+1)/2 connections when the NN has L layers, allowing efficient gradient propagation and feature reuse while substantially reducing the number of parameters. The architecture is characterized by dense blocks and transition layers, which accelerate information flow and parameter reduction.

Vision Transformer (ViT) represents a novel approach to image segmentation by processing images as sequences of patches through self-attention mechanisms (Dosovitskiy et al., 2021). Unlike CNNs, ViT can learn global relationships and create connections between distant regions in the image. ViT has recently demonstrated remarkable performance on large datasets and has become increasingly attractive for complex tumor segmentation tasks (Drury et al., 2025).

DeepLabV3+ is an encoder-decoder semantic segmentation architecture that combines atrous (dilated) convolutions with an effective decoder to capture contextual information at multiple spatial scales (Chen et al., 2018). In OC segmentation and detection, DeepLabV3+ provides accurate tumor localization and boundary delineation by combining high spatial resolution with contextual information.

For a consistent pixel-wise comparison, all nine architectures were implemented within a semantic segmentation framework. U-Net, Attention U-Net, SegNet, and DeepLabV3+ were used in their native encoder-decoder segmentation configurations. For the classification-oriented CNN architectures (VGG16, ResNet50, DenseNet-121, and GoogLeNet), the classification heads were removed, and the convolutional feature-extraction components were used as encoders. The extracted multi-scale feature maps were connected to decoder blocks composed of upsampling and convolutional layers, with skip connections from the corresponding encoder stages where applicable, to restore the spatial resolution of the input and generate pixel-wise segmentation maps. ViT was similarly adapted for segmentation by using transformer-based image representations followed by a segmentation decoder that reconstructed the pixel-level output. The final layer generated the class-specific pixel-wise prediction maps required by the proposed framework.

Before model training, CT intensities were clipped to a fixed range of −150 HU to +250 HU and subsequently linearly normalized to the [0,1] range. This preprocessing step was implemented as direct intensity clipping rather than using a window-center/window-width convention. All axial CT slices were resized to 256 × 256 pixels. Segmentation masks were resized using nearest-neighbor interpolation to preserve discrete class labels. Data augmentation was applied only to the training cohort and included random rotations within ±10°, horizontal flipping, translations of up to 10% of the image dimensions, and random scaling within the range 0.90–1.10. No augmentation was applied to the validation or testing cohorts.

All models were trained using the Adam optimizer with an initial learning rate of 1 × 10^−4^ and a batch size of 8. A combined Dice and categorical cross-entropy loss was used to account for both spatial overlap and pixel-wise class prediction. Class imbalance was addressed by weighting the categorical component according to the relative frequency of the classes in the training cohort. Training was performed for a maximum of 100 epochs. The learning rate was reduced when the validation loss stopped improving, and early stopping was applied with a patience of 15 epochs; the model weights corresponding to the best validation performance were retained. A fixed random seed of 42 was used for data partitioning and model initialization to improve reproducibility. The validation cohort was used exclusively for model selection and ensemble-weight estimation, while the patient-disjoint testing cohort remained unseen until the final evaluation.

For architectures originally developed for image classification, including VGG16, ResNet50, DenseNet-121, and GoogLeNet, ImageNet-pretrained weights were used to initialize the encoder components. The original classification heads were removed and replaced by segmentation decoders. During transfer learning, the pretrained encoder weights were used as initialization and subsequently fine-tuned jointly with the decoder on the TCGA-OV training cohort. For the segmentation-specific architectures, the complete networks were optimized for the present segmentation task under the same training protocol, thereby allowing a consistent comparison among architectures.

### 3.3 Hardware and software specifications

To manipulate high-resolution CT images, medical image preprocessing libraries such as SimpleITK and MONAI, and machine learning frameworks, including TensorFlow and PyTorch, were utilized. Software such as 3D Slicer, Radiomics, or ITK-SNAP was used for image analysis. Image organization, inspection, and part of the preprocessing workflow were performed on a local Acer Aspire C system equipped with an Intel Core i5-1235U processor, 16 GB DDR4 RAM, a 512 GB M.2 NVMe SSD, and Intel Iris Xe integrated graphics. Because this local configuration was not used as the principal accelerator for computationally intensive deep-learning training, neural-network training and evaluation were performed in the Google Colab cloud environment using the available GPU-accelerated runtime. The implementation was developed in Python using TensorFlow/PyTorch-based deep-learning tools, while SimpleITK and MONAI were used for medical-image preprocessing. 3D Slicer was used for segmentation review and refinement, and ITK-SNAP was used for checking the segmentation masks.

### 3.4 Performance indicators

To validate the performance of the models, the following statistical parameters were considered: accuracy, precision, recall, IoU (Intersection over Union), DICE Similarity Coefficient (DSC)/ F1 score, and other indicators such as processing time and number of parameters. For the five-class analysis, the ground-truth mask therefore represents a composite label: the No Tumor class corresponds to pixels outside the anatomical tumor mask, whereas the four tumor classes correspond to pixels inside the tumor mask carrying the molecular subtype assigned at the patient level. Accordingly, class-specific pixel-level metrics quantify agreement with this composite annotation and should not be interpreted as evidence of direct voxel-wise molecular subtype identification from CT images.

In addition to the pooled pixel-level evaluation, a patient-level analysis was performed to account for the non-independence of multiple CT slices originating from the same subject. For each patient in the testing cohort, the segmentation metrics were first calculated using all corresponding test images, yielding one value per metric per patient. Patient-level F1 score (Dice), IoU, precision, and recall were then summarized across the testing cohort using the mean and standard deviation. Thus, each patient contributed equally to the patient-level performance estimate, irrespective of the number of CT slices available for that subject.

To quantify the uncertainty associated with the limited number of independent subjects in the testing cohort, 95% confidence intervals were estimated using non-parametric bootstrap resampling at the patient level. Patients, rather than individual slices or pixels, were used as the resampling units in order to preserve the within-patient correlation structure. For each bootstrap iteration, patients were sampled with replacement from the testing cohort, and the corresponding patient-level performance metric was recalculated. The 2.5th and 97.5th percentiles of the bootstrap distribution were used to define the 95% confidence interval.

Comparisons between the individual models and the successive fusion configurations were performed at the patient level. Because the same patients were evaluated by each competing configuration, paired patient-level measurements were used. Given the limited size of the testing cohort and without assuming normality of the performance distributions, paired comparisons were assessed using the two-sided Wilcoxon signed-rank test. Statistical significance was defined as *p* < 0.05. In addition, 95% confidence intervals for the differences in performance were estimated by non-parametric bootstrap resampling at the patient level. Patients, rather than individual slices or pixels, were used as the resampling units to preserve within-patient dependence

The evaluation framework was intentionally kept identical for all individual networks and fusion configurations to enable a direct comparison under the same experimental conditions. The principal comparative indicators were F1/Dice, recall, and precision, while IoU and accuracy were used as complementary segmentation measures. Although additional boundary- and volume-based measures such as HD95, average surface distance, lesion-wise sensitivity, and tumor-volume error may provide further information regarding clinical segmentation quality, these measures were outside the predefined evaluation protocol of the present comparative fusion study and were therefore not retrospectively introduced after model evaluation.

Particular caution was applied when interpreting overall pixel-level performance because the No Tumor class represents the background and may contain a substantially larger number of pixels than the individual tumor classes. Consequently, overall accuracy was not interpreted independently as evidence of tumor-segmentation quality. Greater emphasis was placed on class-specific F1/Dice, recall, precision, and IoU, which provide a more informative assessment of performance for the individual tumor-related classes.

For the patient-level uncertainty analysis, 10,000 non-parametric bootstrap resamples were generated with replacement, using the patient rather than the individual CT slice or pixel as the resampling unit. The 2.5th and 97.5th percentiles of the resulting bootstrap distributions defined the 95% confidence intervals. Paired comparisons between configurations were performed on the corresponding patient-level metric values using two-sided Wilcoxon signed-rank tests, with p < 0.05 considered statistically significant.

Table 1 contains the formulas depending on the values of *TP* (True Positives), *FP* (False Positives), *FN* (False Negatives, and *TN* (True Negatives) for each statistical parameter.

**Table 1:** Performance indicators.

Parameter (%)	Formula
Accuracy (*ACC*)	*ACC* = $\frac{TP\;+\;TN}{\;TP\;+\;TN\;+\;FP\;+\;FN\;}\times 100$
Precision (*P*)	*P* = $\frac{TP\;}{TP\;+\;FP\;}\times 100$
Recall (*R*)	*R* = $\frac{TP\;}{\;TP\;+\;FN}\times 100$
*F*1 Score (*F*1)	*F1* = $\frac{2\;\times \;TP\;}{2\;\times \;TP\;+\;FP\;+\;FN}\times 100$
*IoU*	*IoU* = $\frac{TP}{\;TP\;+\;FP\;+\;FN}\times 100$

### 3.5 Comparative analysis and selection of neural networks

The individual NN models were empirically selected from a literature review: NN1 - Attention U-Net, NN2 - DeepLabV3+, NN3 – ViT, NN4 - VGG16, NN5 - SegNet, NN6 - ResNet50, NN7 - U-Net, NN8 - DenseNet-121, NN9 – GoogLeNet. The performance data are presented in Section 5. The previous NN architectures were implemented using transfer-learning methods to generate pixel-wise predictions for the composite five-class target representation (No Tumor – class C1, Differentiated – class C2, Proliferative – class C3, Mesenchymal – class C4, and Immunoreactive – class C5) from CT images of the TCGA-OV dataset. The implementation of the system for ovarian tumor detection and segmentation consists of two essential phases: (a) Performing a test of individual NNs to select the system components by choosing the top 3 best-performing NNs according to F1 score, recall, and precision. (b) Developing and testing an ensemble model with two fusion stages.

In Figure 3, the conceptual model of the proposed dual decision fusion system is shown. First, based on the methodology for evaluating the performance of each proposed NN for tumor segmentation in each class, the top three NNs are selected from a general ranking. According to the results in Section 4, Attention U-Net, DeepLabV3+, and ViT were selected. For the first level of decision fusion, 4 ensembles were considered: E1 (voting with binary decision, 0 for the non-class and 1 for the class), E2, E3, and E4, with decisions based on weighted scores of the considered metrics (for E2 – F1 score, for E3 – recall, and for E4 – precision). Depending on the performances obtained, weight tables were established for the ensembles based on weighted scores, corresponding to the NNs and classes (Section 4). The second level of decision fusion combines the decisions of the ensembles from the first level to form the global system (GS) based on the weighted score, considering only the F1 score (the most representative).

**Figure 3:**
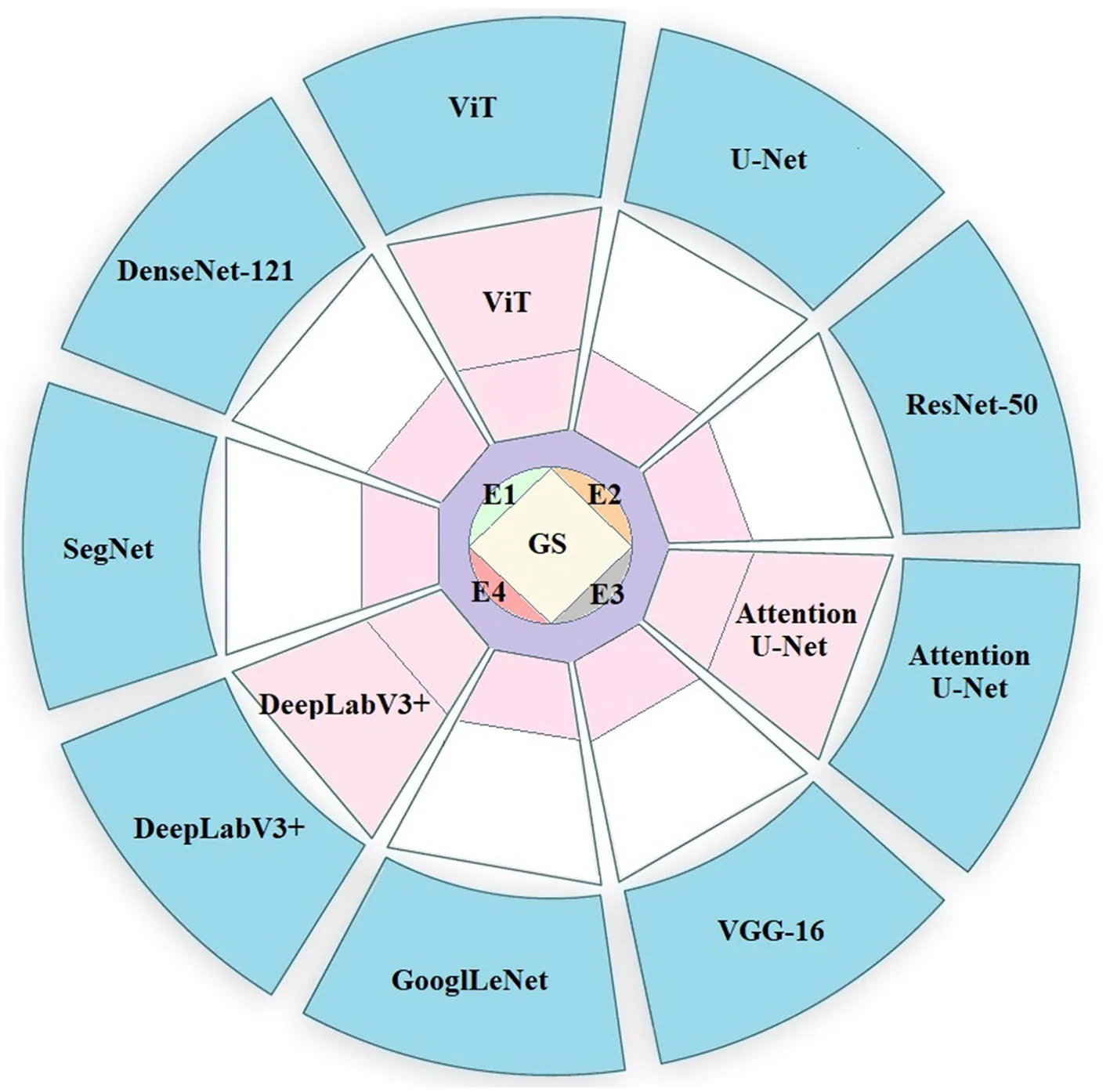
The conceptual model of the dual decision fusion system.

For the proposed system, the building workflow from Figure 4 is followed. The meaning of the notations is as follows: IPP – image preprocessing module; NN1, NN2, …, NN9 – neural networks to be compared and selected; MPC – model performance comparison; SNN1, SNN2, SNN3 – selected neural networks to build the ensemble modules; WMN – weight module of selected networks; E1, E2, E3, E4 – ensemble modules of neural networks (first level of fusion); WME – weight module of ensembles; GS – global system (second level of fusion); C1, C2, C3, C4, C5 – classes in which the segmented tumor should be classified. In the learning phase, the first fusion level is created by following the steps below. Step 1 – Retrieving the image from the database. Step 2 – Preprocessing (primary image processing) for noise removal, contrast enhancement, and normalization (IPP module). Step 3 – Calculating the performance indicators for the nine considered networks, comparing them, and selecting the three most efficient networks to form the ensemble modules at the first fusion level. Step 4 – Establishing the weights (WMN) associated with the individual networks for decision fusion. Step 5 – Choosing the assembly modules and establishing the operating equations. In the validation phase, the weights are determined for creating the second fusion level – the global system.

**Figure 4:**
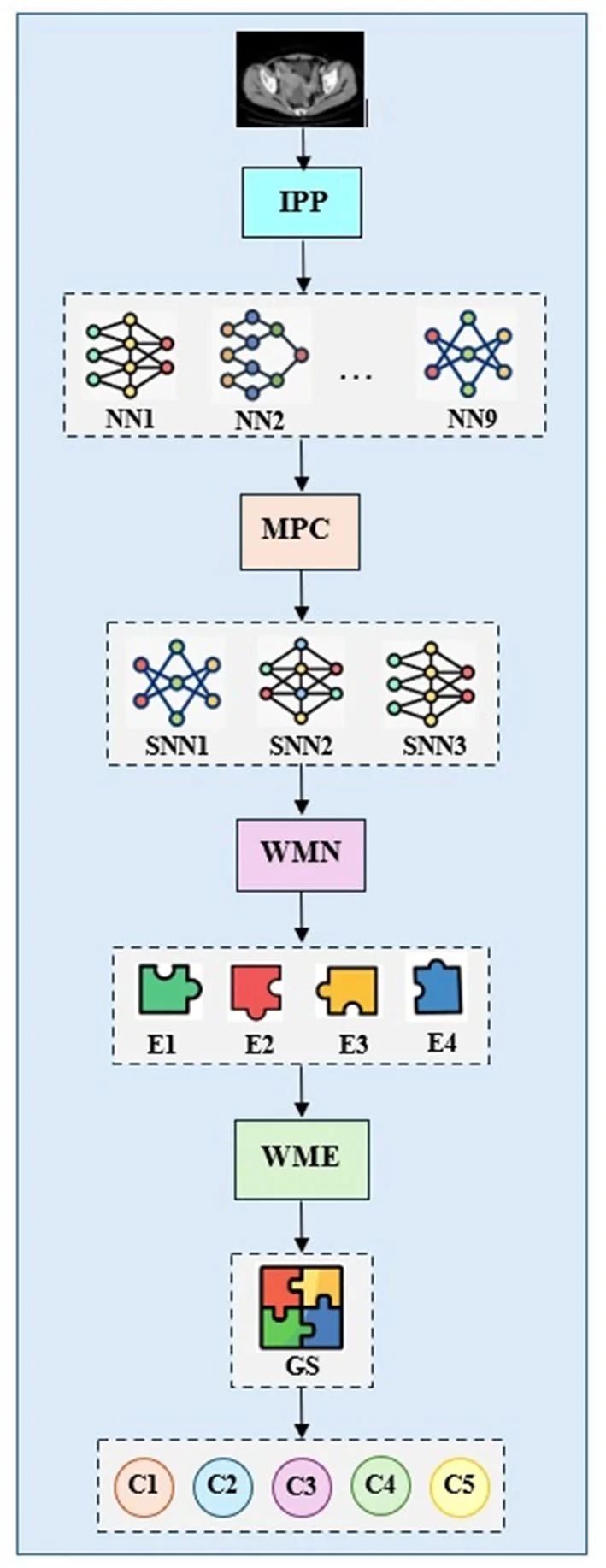
The workflow for building the global system.

A comparative component-selection analysis was performed to evaluate the candidate neural networks and identify the best-performing complementary models for subsequent ensemble construction. For individual network selection, each pair (network *NNi* and class *Cj*) is assigned a position (*Pos*) in a ranking based on its respective performance (1 being the best position): *Pos*__*F*_1_ – for *F*1 score, *Pos*_*R*_ – for Recall, and *Pos*_*P*_ – for Precision. A score *Si* is established for each *NNi* network acording with Equation 1:


$$S_{i}=\overset{5}{\underset{j=1}{\sum }}[Pos_{F1}(NN_{i},C_{j})+Pos_{R}(NN_{i},C_{j})+Pos_{P}(NN_{i},C_{j})],\;i=1,2,\ldots ,9.$$ (1)

The smallest *Si* determines the best *NNi*. Thus, we empirically determined to select three NNs (SNN1, SNN2, and SNN3) by considering the first three lowest scores (the first three positions in the ranking). For the first level of decision fusion, the three best individual models were selected based on a binary decision and weighted scores for *F*1, recall, and precision. The selected NNs, based on results from Section 4, were Attention U-Net, DeepLabV3+, and ViT.

Network selection and ensemble-weight estimation were performed exclusively using the validation cohort; the independent testing cohort was not used at any stage of model selection, weight estimation, or ensemble construction. The three component networks were first selected according to their validation F1-score, recall, and precision rankings. Subsequently, class-specific performance values from the validation cohort were used to determine the weights of the first-level ensembles. Therefore, although the testing cohort remained strictly independent, model selection and weight estimation were both dependent on the same validation population.

### 3.6 Global system implementation

To classify a pixel *x* into a class *C*(j), by the ensemble Ek (*k* = 1, 2, 3, 4), or the Global System (GS), a score attached to the classes for this ensemble, *SC*_*k*_(*j*), or to the global system *SGS*(j), *j* = 1, 2, 3, 4, 5, was considered (2), (3), (5), and the maximum score (4), (6) was retained.

For E1, the score attached to the class *C(j), SC*_1_*(j)*, is calculated by Equation 2:


$$\begin{array}{l} SC_{1}\left(j\right)=\overset{3}{\underset{i=1}{\sum }}d_{i}\left(j\right)\cdot p_{i}\left(j\right),\;j=1,\;2,\;3,\;4,\;5 \end{array}$$ (2)

where *d*_*i*_(*j*) is a binary decision of *NNi* (1 or 0) and *p*_*i*_(*j*) is the probability that *x*∈*C*(*j*).

For En, *n* = 2, 3, 4, the score attached to the class *C(j)* is *SC*_*n*_*(j)* calculated by Equation 3:


$$\begin{array}{l} SC_{n}\left(j\right)=\overset{3}{\underset{i=1}{\sum }}w_{i}^{n}\left(j\right)\cdot p_{i}\left(j\right),\;j=1,\;2,\;3,\;4,\;5 \end{array}$$ (3)

where $w_{i}^{n}\left(j\right)$ is the weight for the *NNi* network and class *C(j)*, and *p*_*i*_(*j*) is the probability expressed by *NNi* that *x*∈*C*(*j*).

Then Ek, *k* = 1, 2, 3, 4 establishes that *x*∈*C*(*j*_0_) if requirement (6) is met:


$$\begin{array}{l} SC_{k}\left(j_{0}\right)=\max\left\{SC_{k}\left(j\right),\;j=1,\;2,\;3,\;4,\;5\right\}. \end{array}$$ (4)

Similarly, for the global *GS* system, the score evaluated for the class *C(j)* is calculated by Equation 5:


$$\begin{array}{l} SGS\left(j\right)=\;\overset{4}{\underset{i=1}{\sum }}SC_{i}\left(j\right)\cdot WE_{i}\left(j\right),\;j=1,\;2,\;3,\;4,\;5 \end{array}$$ (5)

where *WE*_*i*_(*j*) is the weight associated with the *E*i ensemble and class *C(j)*. Then GS establishes that *x*∈*C*(*j*_0_) if requirement (4) is met:


$$SGS(j_{0})=\max\{SGS(j)\},\;j=1,\;2,\;3,\;4,\;5\}.$$ (6)

Weights for ensembles E2, E3, E4, and GS are considered for each input and class (Tables 2, 3). Thus, for E2, the weights are the *F*1 indicators; for E3, the weights are the recall; for E4, the weights are the precision; and for GS, the weights are the *F*1 indicators associated with the first-level ensembles and classes. Actual values of these weights are calculated in the “Experimental Results” section. R2.13.

**Table 2:** Formal weights for ensembles E2, E3, and E4.

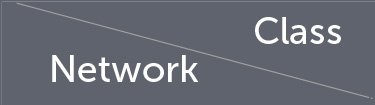	C1	C2	C3	C4	C5
E2					
NN1	$W_{1}^{2}$(1)	$W_{1}^{2}$(2)	$W_{1}^{2}$(3)	$W_{1}^{2}$(4)	$W_{1}^{2}$(5)
NN2	$W_{2}^{2}$(1)	$W_{2}^{2}$(2)	$W_{2}^{2}$(3)	$W_{2}^{2}$(4)	$W_{2}^{2}$(5)
NN3	$W_{3}^{2}$(1)	$W_{3}^{2}$(2)	$W_{3}^{2}$(3)	$W_{3}^{2}$(4)	$W_{3}^{2}$(5)
**E3**					
NN1	$W_{1}^{3}$(1)	$W_{1}^{3}$(2)	$W_{1}^{3}$(3)	$W_{1}^{3}$(4)	$W_{1}^{3}$(5)
NN2	$W_{2}^{3}$(1)	$W_{2}^{3}$(2)	$W_{2}^{3}$(3)	$W_{2}^{3}$(4)	$W_{2}^{3}$(5)
NN3	$W_{3}^{3}$(1)	$W_{3}^{3}$(2)	$W_{3}^{3}$(3)	$W_{3}^{3}$(4)	$W_{3}^{3}$(5)
**E4**					
NN1	$W_{1}^{4}$(1)	$W_{1}^{4}$(2)	$W_{1}^{4}$(3)	$W_{1}^{4}$(4)	$W_{1}^{4}$(5)
NN2	$W_{2}^{4}$(1)	$W_{2}^{4}$(2)	$W_{2}^{4}$(3)	$W_{2}^{4}$(4)	$W_{2}^{4}$(5)
NN3	$W_{3}^{4}$(1)	$W_{3}^{4}$(2)	$W_{3}^{4}$(3)	$W_{3}^{4}$(4)	$W_{3}^{4}$(5)

**Table 3:** Formal weights for the global system.

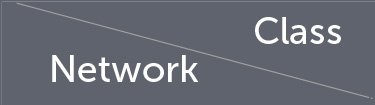	C1	C2	C3	C4	C5
**E1**	WE1(1)	WE1(2)	WE1(3)	WE1(4)	WE1(5)
**E2**	WE2(1)	WE2(2)	WE2(3)	WE2(4)	WE2(5)
**E3**	WE3(1)	WE3(2)	WE3(3)	WE3(4)	WE3(5)
**E4**	WE4(1)	WE4(2)	WE4(3)	WE4(4)	WE4(5)

The weights for the component modules of the proposed system were established during the learning and validation stages. The operation (testing) stage follows based on Equations (2) ÷ (6), summarized in Figure 5. The significance of the notations in the figure is as follows: IPP – image preprocessing module; SNN1, SNN2, SNN3 – selected neural networks; *p*_*i*_(j) – probability associated with the SNNi decision for the Cj class; *d*_i_(j) – SNNi decision (vote) of Cj class for E1; $w_{i}^{2}\left(j\right)$ – SNNi weight of Cj class for E2; $w_{i}^{3}\left(j\right)$ – SNNi weight of Cj class for E3, $w_{i}^{4}\left(j\right)$ – SNNi weight of Cj class for E4; E1, E2, E3, E4 – ensemble modules; *SC*_1_(*j*) – E1 decision for Cj class; *SC*_2_(*j*) – E2 decision for Cj class; $SC_{3}^{j}$ – E3 decision for Cj class; $SC_{4}^{j}$ – E4 decision for Cj class; WE1(j) – weight of Cj class for E1 ensemble; WE2(j) – weight of Cj class for E2 ensemble; WE3(j) – weight of Cj class for E3 ensemble; WE4(j) – weight of Cj class for E4 ensemble; GS – global system; C1, C2, C3, C4, C5 – tumor classes for classification.

**Figure 5:**
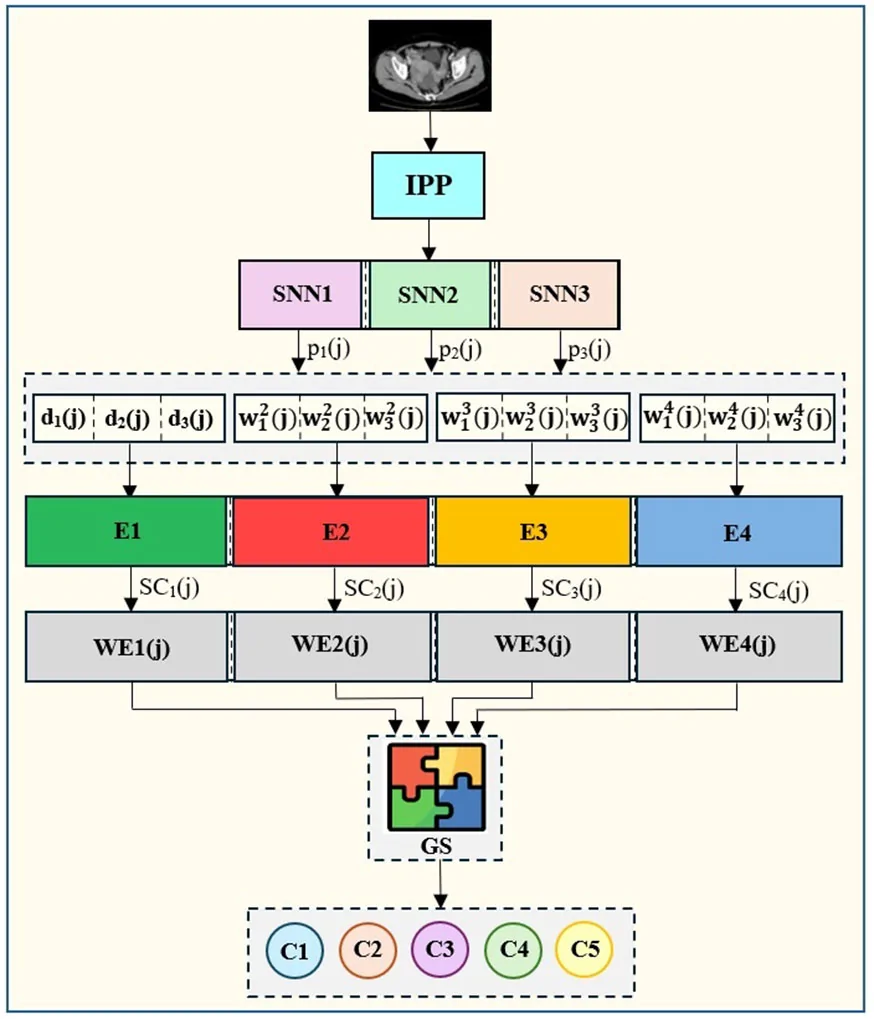
The operating mode of the global system.

First, the IPP module preprocesses the input image. The primary neural networks SNNi (*i* = 1, 2, 3) classify each image pixel into one of the classes *Cj* (*j* = 1, 2, 3, 4, 5) with a probability *p*_*i*_(*j*). The results of the primary classification of the pixels, adjusted with the previously mentioned weights $w_{i}^{n}\left(j\right),\;$(*n* = 2, 3, 4) or the factors *d*_*i*_(*j*), are evaluated by the ensembles Ek (*k* = 1, 2, 3, 4) and classified, in the first fusion stage, into the classes *C*j with the scores *SCk*(*j*). In the second fusion stage, the final classification of the pixel is made based on the maximum score obtained through relations (5) and (6) and considering the weights previously assigned to the *WEk*(*j*) ensembles and the *SCk*(*j*) scores.

The weighting procedure was deterministic and based exclusively on validation-set performance; no weights were optimized against the independent testing cohort. Nevertheless, because the same validation cohort contributed both to component-model selection and to subsequent class-specific weight estimation, the resulting ensemble may be susceptible to validation-set optimization bias, particularly given the limited number of independent patients. This issue is considered when interpreting the final test performance.

## 4 Experimental results

According to the patient-level partitioning strategy, the TCGA-OV dataset was divided into training (70%), validation (20%), and testing (10%) cohorts across the 143 subjects. The corresponding image counts reflect this subject-level allocation, and class proportions were preserved across subsets. All performance metrics were computed on the patient-disjoint testing cohort to ensure unbiased segmentation evaluation.

For each of the 9 networks, the learning loss and accuracy were evaluated as a function of the number of epochs (Figure 6). For 100 epochs, the results were similar (accuracy greater than 0.85 and loss function around 0.5).

**Figure 6:**
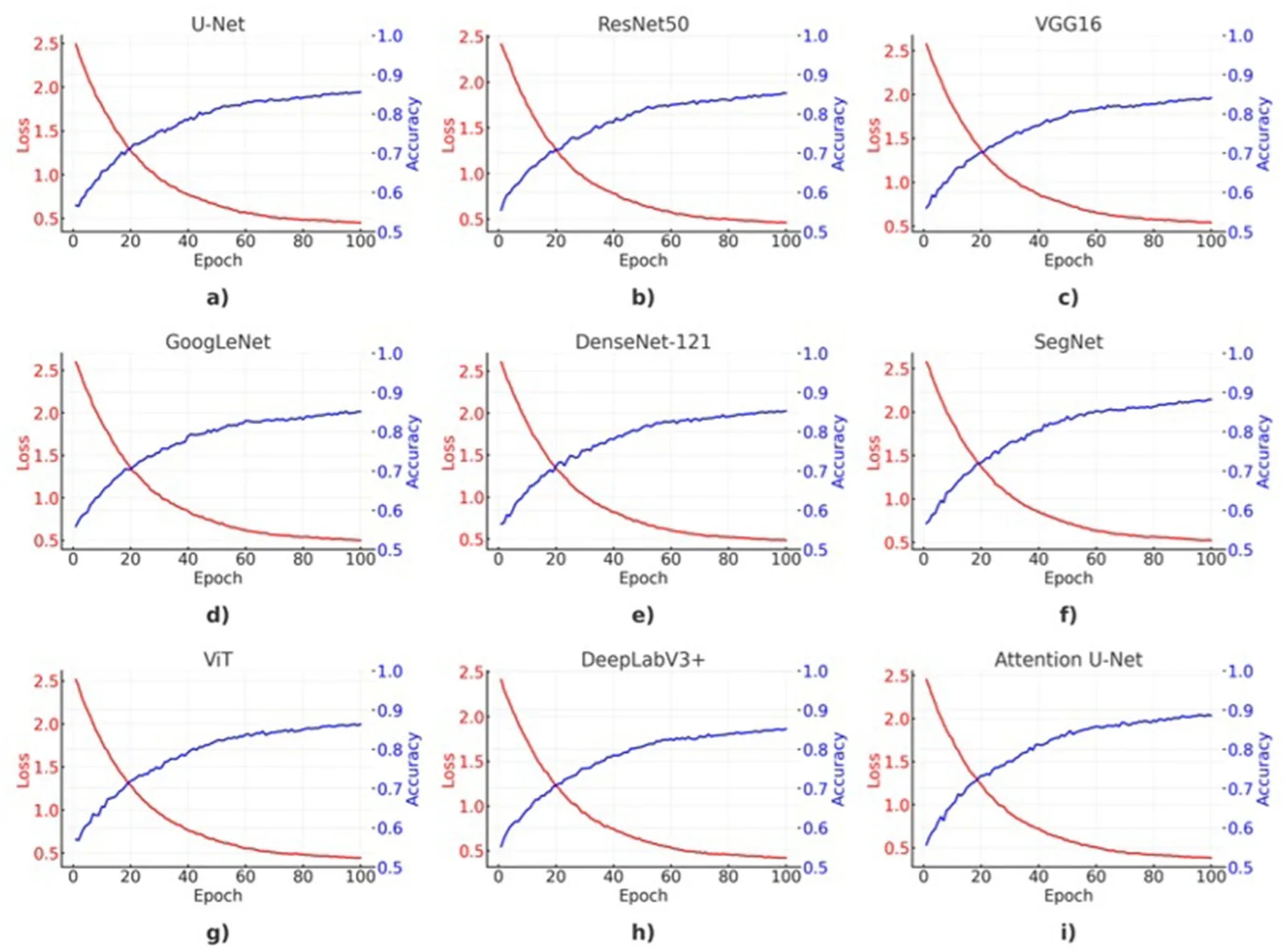
Loss function (red) and accuracy (blue) through the training for each neural network. **(a)** U-Net, **(b)** ResNet50, **(c)** VGG16, **(d)** GoogLeNet, **(e)** DenseNet-121, **(f)** SegNet, **(g)** ViT, **(h)** DepLabV3+, **(i)** Attention U-Net

The results of the network classification and selection, based on the three metrics - *F*1 score (Table 4), *Recall* (Table 5), and *Precision* (Table 6) - are presented. A comparative ranking procedure was performed to select the neural networks included in the first ensemble level. A position Pj of the performance considered for each class Cj was established (position 1 is the best) for each NN. At the end of the line corresponding to the network, the sum of the positions for the 5 classes was calculated. The lowest sum indicates the best network for the respective performance. At the end of Table 6, the total sums (Sum) and the corresponding final positions (Poz) were calculated. Position 1 corresponds to the minimum sum. The selection process chooses the first three networks (positions 1, 2, and 3). The results in Table 7, marked in gray, show S2 = 29 (for the best network, NN2), S1 = 30 (for the network NN1), and S3 = 31 (for the network NN3). Thus, Attention U-Net (SNN1), DeepLabV3+ (SNN2), and ViT (SNN3) are the selected networks for the first level of fusion.

**Table 4:** Individual network performance obtained on the validation set based on the F1 score.

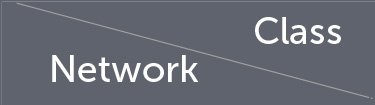	F1 (%)										
	C1	P1	C2	P2	C3	P3	C4	P4	C5	P5	Sum
**NN1**	90.89	1	87.19	3	88.46	3	90.78	1	90.01	1	9
**NN2**	89.10	2	89.89	1	88.65	2	89.28	2	84.92	3	10
**NN3**	88.59	3	88.73	2	93.36	1	86.21	3	86.56	2	11
**NN4**	81.30	9	80.53	9	78.92	9	76.83	9	80.13	9	45
**NN5**	85.48	7	83.49	8	87.79	4	83.99	5	83.64	6	30
**NN6**	86.19	4	85.43	6	84.44	6	85.15	4	83.70	5	25
**NN7**	84.06	8	86.14	4	84.14	8	83.50	6	83.42	8	34
**NN8**	86.19	5	83.93	7	85.07	5	81.69	8	84.55	4	29
**NN9**	86.18	6	85.63	5	84.22	7	82.86	7	83.38	7	32

**Table 5:** Individual network performance obtained on the validation set based on the Recall.

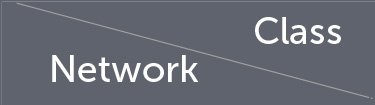	Recall (%)										
	C1	P1	C2	P2	C3	P3	C4	P4	C5	P5	Sum
**NN1**	90.28	2	87.49	2	90.09	2	89.76	1	94.06	1	8
**NN2**	86.38	3	89.25	1	88.73	3	84.69	2	83.51	3	12
**NN3**	91.28	1	85.44	3	93.71	1	83.13	3	86.81	2	10
**NN4**	77.07	9	75.04	9	72.34	9	69.48	9	75.82	9	45
**NN5**	86.35	8	85.42	4	88.71	4	83.11	5	83.48	7	28
**NN6**	86.36	4	84.04	7	85.94	5	83.10	7	83.49	4	27
**NN7**	86.35	6	85.41	5	82.97	8	83.11	6	83.49	5	30
**NN8**	86.36	5	81.88	8	83.34	7	83.12	4	82.94	8	32
**NN9**	86.35	7	84.79	6	84.92	6	81.50	8	83.48	6	33

**Table 6:** Individual network performance obtained on the validation set based on Precision and the NN selection for GS.

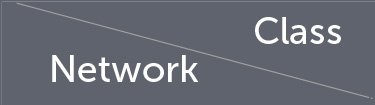	Precision (%)											Selection	
	**C1**	**P1**	**C2**	**P2**	**C3**	**P3**	**C4**	**P4**	**C5**	**P5**	**Sum**	**Tot**	**Poz**
**NN1**	91.49	2	86.90	3	86.89	3	91.82	2	86.29	3	13	30	**2**
**NN2**	92.01	1	90.53	2	88.57	2	94.40	1	86.37	1	7	29	**1**
**NN3**	86.05	3	92.28	1	93.01	1	89.53	3	86.31	2	10	31	**3**
**NN4**	86.02	6	86.88	4	86.81	6	85.91	5	84.97	5	26	116	9
**NN5**	84.62	8	81.65	9	86.88	4	84.89	6	83.79	7	34	92	5
**NN6**	86.03	4	86.87	5	82.99	9	87.30	4	83.91	6	28	80	4
**NN7**	81.90	9	86.87	6	85.34	7	83.89	8	83.35	8	38	102	7
**NN8**	86.03	5	86.09	8	86.87	5	80.31	9	86.22	4	31	92	6
**NN9**	86.01	7	86.49	7	83.52	8	84.26	7	83.29	9	38	103	8

^*^Bold values means the best three values of position for individual networks tested.

**Table 7:** Weight values for ensembles.

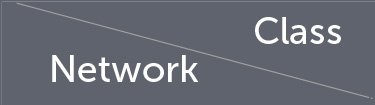	C1	C2	C3	C4	C5
E2					
**SNN1**	0.9089	0.8719	0.8846	0.9078	0.9001
**SNN2**	0.8910	0.8989	0.8865	0.8928	0.8492
**SNN3**	0.8859	0.8873	0.9336	0.8621	0.8656
**E3**					
**SNN1**	0.9028	0.8749	0.9009	0.8976	0.9406
**SNN2**	0.8638	0.8925	0.8873	0.8469	0.8351
**SNN3**	0.9128	0.8544	0.9371	0.8313	0.8681
**E4**					
**SNN1**	0.9149	0.869	0.8689	0.9182	0.8629
**SNN2**	0.9201	0.9053	0.8857	0.9440	0.8637
**SNN3**	0.8605	0.9228	0.9301	0.8953	0.8631

According to Section 3.6 (Table 2), the weights associated with networks SNN1, SNN2, and SNN3 for ensembles E2 (based on *F*1), E3 (based on *Recall*), and E4 (based on *Precision*) are presented in Table 7 (corresponding to the values of the performance indicators in Tables 4–6).

With the weights associated with E2, E3, and E4, for the ensembles of the first level of fusion, F1 Score, recall, and precision were obtained (Table 8). As weights (Table 3) for the information from the E1, E2, E3, and E4 ensembles for the global system (WE1, WE2, WE3, and WE4), the values of the *F*1 indicator from Table 8 were taken because *F*1 combines information from precision and recall. The weights for GS are presented in Table 9. Examples of confusion matrices for pixel classification in two images in the testing phase of the global system are presented in Figure 7.

**Table 8:** Performance obtained on the validation set on ensembles E1, E2, E3, and E4.

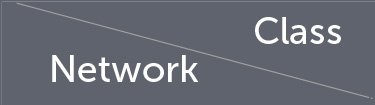	C1	C2	C3	C4	C5
F1 (%)					
**E1**	90.84	89.60	92.66	90.51	89.04
**E2**	91.34	89.83	91.95	90.33	88.97
**E3**	90.77	89.74	92.15	90.68	89.43
**E4**	90.84	90.55	92.13	90.00	89.05
**Recall (%)**					
**E1**	90.76	88.30	93.06	87.36	89.97
**E2**	91.35	88.55	92.76	87.10	90.01
**E3**	90.40	88.19	92.46	87.91	90.26
**E4**	90.68	89.68	93.03	87.30	90.03
**Precision (%)**					
**E1**	90.93	0.90.94	92.25	93.89	88.14
**E2**	91.33	91.15	91.17	93.81	87.94
**E3**	91.15	91.35	91.83	93.62	88.62
**E4**	91.00	91.43	91.25	92.88	88.10

**Table 9:** Weight values for the global system.

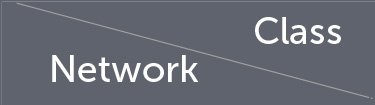	C1	C2	C3	C4	C5
**E1**	0.9084	0.8960	0.9266	0.9051	0.8904
**E2**	0.9134	0.8983	0.9195	0.9033	0.8897
**E3**	0.9077	0.8974	0.9215	0.9068	0.8943
**E4**	0.9084	0.9055	0.9213	0.9000	0.8905

**Figure 7:**
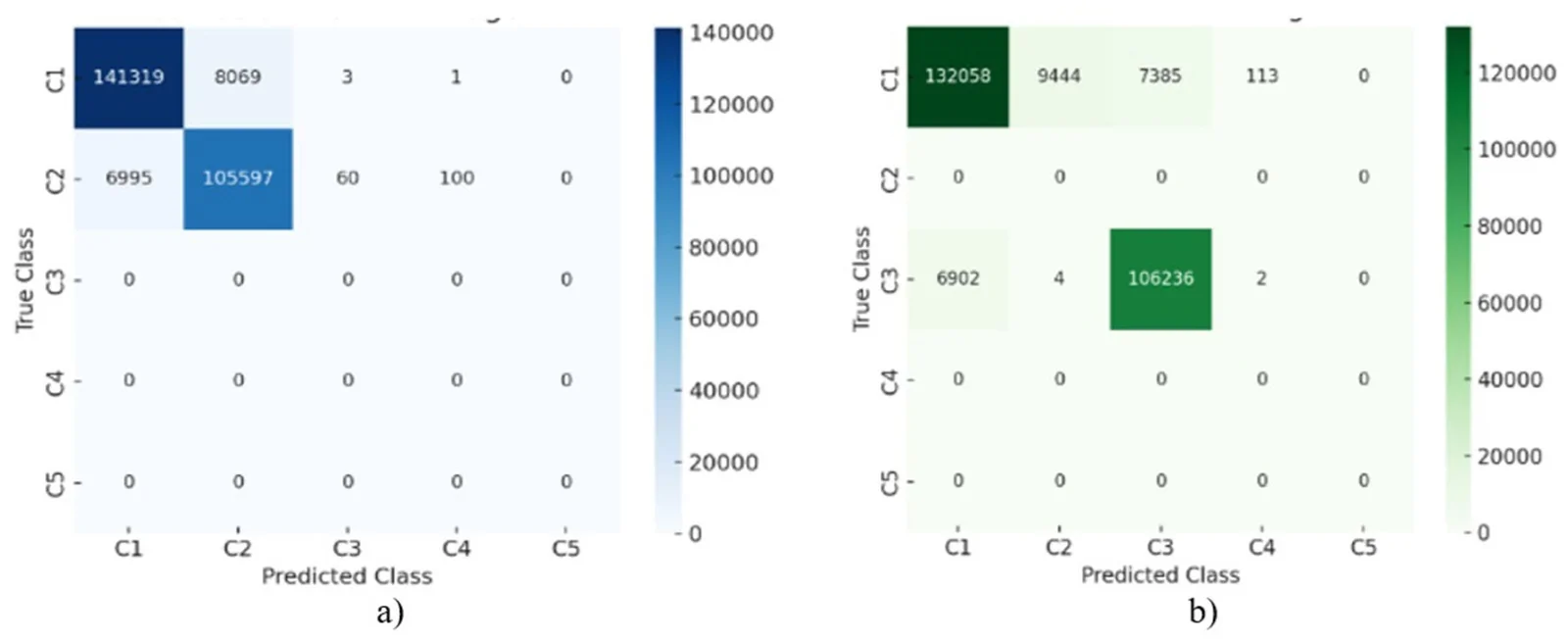
Confusion matrix for two images **(a)** and **(b)** on the testing set (global system).

Figure 7 presents representative image-level confusion matrices as qualitative examples of the class-specific prediction patterns. These examples are provided for visualization purposes and should not be interpreted as a substitute for the quantitative evaluation reported for the complete patient-disjoint testing cohort. The matrices are provided for qualitative illustration of class-specific prediction patterns and do not represent an aggregate confusion matrix for the complete test cohort.

To evaluate and compare the overall performance of the final system, the *F*1, Recall, and Precision indicators were calculated for the individual SNNs (SNN1, SNN2, and SNN3), the first-level ensembles (E1, E2, E3, and E4), and the global system (GS) (Table 10). The performances were calculated for each class and as an average value using the test dataset. It is observed that the performances of the first-level ensembles were better than those of the individual NNs, and the performances of the global system (level two) were better than those of the first-level ensembles.

**Table 10:** Performance obtained on the test set.

	C1	C2	C3	C4	C5	Average
F1 (%)						
**NN1**	87.65	84.91	85.41	88.26	86.70	86.59
**NN2**	87.09	88.38	85.56	86.49	82.51	86.01
**NN3**	85.87	86.62	90.74	83.99	83.92	86.23
**E1**	90.79	89.55	92.60	90.46	88.91	90.46
**E2**	91.29	89.78	91.91	90.28	88.74	90.40
**E3**	90.72	89.69	92.09	90.63	89.35	90.50
**E4**	90.79	90.45	92.08	89.95	88.76	90.41
**GS**	**94.00**	**93.10**	**93.70**	**92.85**	**94.00**	**93.53**
**Recall (%)**						
**NN1**	87.19	86.66	88.07	87.42	90.83	88.03
**NN2**	83.16	88.80	85.72	81.68	80.91	84.05
**NN3**	88.82	82.68	91.01	80.50	84.34	85.47
**E1**	90.71	88.25	93.01	87.31	89.92	89.84
**E2**	91.30	88.50	92.71	87.05	89.96	89.90
**E3**	90.35	88.14	92.41	87.86	90.21	89.79
**E4**	90.63	89.54	92.98	87.25	89.98	90.08
**GS**	**93.80**	**93.20**	**93.30**	**92.40**	**94.20**	**93.38**
**Precision (%)**						
**NN1**	88.11	83.23	82.91	89.12	82.93	85.26
**NN2**	91.41	87.96	85.40	91.90	84.17	88.17
**NN3**	83.11	90.95	90.47	87.80	83.50	87.17
**E1**	90.88	90.89	92.20	93.84	87.92	91.15
**E2**	91.28	91.10	91.12	**93.76**	87.56	90.96
**E3**	91.10	91.30	91.78	93.57	88.51	91.25
**E4**	90.95	91.38	91.20	92.83	87.57	90.79
**GS**	**94.20**	**93.00**	**94.10**	**93.30**	**93.80**	**93.68**

^*^Bold values means the best values of performances F1, Recall, and Precision for different classes.

In Figure 8, an example of an image from the database shows where the tumor is segmented (green color) using the 8 analyzed models: (a) original image with tumor and ground truth segmentation; (b) tumor segmented with Attention U-Net; (c) tumor segmented with DeepLabV3+; (d) tumor segmented with ViT; (e) tumor segmented with E1; (f) tumor segmented with E2; (g) tumor segmented with E3; (h) tumor segmented with E4; (i) tumor segmented with GS (Global System).

**Figure 8:**
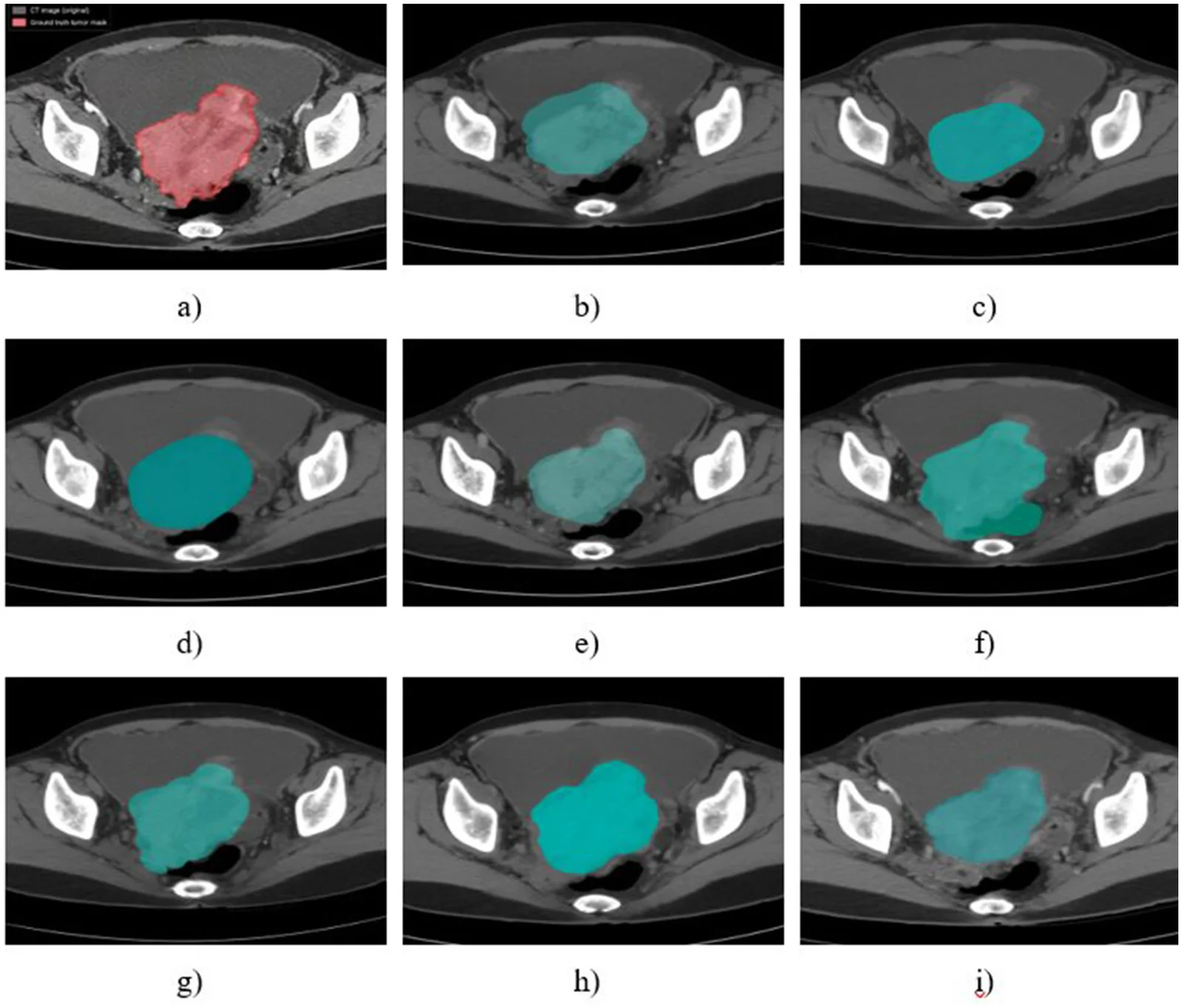
Example of segmentation for a differentiated tumor image, **(a)** Ground Truth image with tumor segmented (dataset used); **(b)** Tumor segmented with Attention U-Net; **(c)** Tumor segmented with DeepLabV3+; **(d)** Tumor segmented with ViT; **(e)** Tumor segmented with E1; **(f)** Tumor segmented with E2; **(g)** Tumor segmented with E3; **(h)** Tumor segmented with E4; **(i)** Tumor segmented with GS (Global System).

Patient-level analysis of the independent test cohort confirmed the performance observed in the pooled segmentation evaluation. The Global System achieved a mean patient-level F1/Dice score of 93.5 ± 2.4% (95% bootstrap CI: 92.2–94.7%), compared with 91.8 ± 3.0% (95% CI: 90.2–93.2%) for Attention U-Net, 91.5 ± 3.2% (95% CI: 89.8–93.0%) for DeepLabV3+, and 92.1 ± 2.8% (95% CI: 90.6–93.4%) for ViT. The corresponding patient-level precision and recall of the Global System were 93.7 ± 2.3% (95% CI: 92.5–94.8%) and 93.4 ± 2.6% (95% CI: 92.0–94.6%), respectively. In paired patient-level comparisons of F1/Dice, the Global System differed from Attention U-Net (*p* = 0.012), DeepLabV3+ (*p* = 0.008), and ViT (*p* = 0.021). Bootstrap 95% confidence intervals for the corresponding paired F1/Dice differences were 0.5–2.9, 0.7–3.3, and 0.3–2.5 percentage points, respectively. All confidence intervals were estimated using 10,000 patient-level bootstrap resamples.

## 5 Discussions

The performance evaluation should be interpreted in light of the construction of the five-class target representation. The No Tumor class corresponds to the background outside the anatomically annotated tumor region, whereas the Differentiated, Immunoreactive, Mesenchymal, and Proliferative classes are patient-level molecular subtype labels mapped onto the corresponding tumor masks. Consequently, the proposed framework primarily evaluates spatial tumor segmentation together with the reproduction of subtype-derived labels within the segmented tumor regions; it does not constitute an independent voxel-wise molecular subtype prediction task. The reported pixel-level F1 score, recall, and precision therefore measure agreement with the composite ground-truth representation. This distinction is important because CT imaging alone cannot establish transcriptomic heterogeneity at voxel level without additional biological or molecular measurements.

From a conceptual perspective, a stricter alternative would be to formulate the problem as two sequential tasks: first, anatomical tumor-vs.-background segmentation and, second, molecular subtype classification at the tumor or patient level. Such a formulation would avoid interpreting propagation of a patient-level molecular label as spatially resolved molecular ground truth. The present study retains the five-class formulation as a computational representation of the available anatomical and molecular labels, while explicitly acknowledging this distinction in the interpretation of the results.

Molecular subtype labels were incorporated through patient-level mapping rather than direct voxel-wise imaging differentiation, reflecting current limitations of CT-based visualization (Huang et al., 2025). Despite the patient-disjoint design, the relatively small number of independent subjects remains an important limitation. In particular, the testing cohort contains only a limited number of patients, and the large number of image slices and pixels should not be interpreted as an equivalent increase in the number of independent observations. Patient-level performance estimates and bootstrap confidence intervals were therefore included to provide a more conservative assessment of model performance. Nevertheless, validation in a larger independent external cohort and repeated patient-level cross-validation are warranted to assess the robustness and generalizability of the proposed framework.

Because multiple CT images were available for individual subjects, the patient-level analysis was considered complementary to the pooled pixel-level evaluation. The patient-level results provide a more conservative estimate of segmentation performance because each subject contributes equally to the summary statistics, independently of the number of available slices. The corresponding bootstrap confidence intervals additionally quantify the uncertainty associated with the limited size of the independent testing cohort.

The evaluation protocol also has limitations regarding the range of segmentation-specific metrics considered. The present study primarily focused on overlap- and classification-based measures to compare individual neural networks and successive fusion configurations under identical conditions. Boundary-based measures such as HD95 and average surface distance, as well as lesion-level sensitivity and tumor-volume error, were not included in the original experimental protocol. Their inclusion in future prospective validation would provide complementary information regarding boundary accuracy, lesion detection, and volumetric agreement. Furthermore, because the No Tumor class represents a comparatively large background region, overall pixel-level metrics should be interpreted together with class-specific performance rather than as standalone indicators of tumor segmentation quality.

The present study was designed as a retrospective methodological evaluation using a publicly available research dataset and should not be interpreted as a clinical validation study. No prospective clinical testing, multi-institutional external validation, formal comparison with expert radiologist performance, or dedicated calibration and uncertainty analysis was performed. Consequently, the reported results primarily demonstrate technical feasibility within the investigated dataset. Independent external validation, reader studies, and prospective clinical evaluation are required before considering the proposed framework for clinical decision-support applications

The main contributions of the article refer to the following aspects:

- Consideration of two levels of decision fusion: the first consisting of the fusion of the decisions of the selected individual networks, and the second consisting of the fusion of the decisions of the ensembles in the first level.
- Choice of networks for the formation of ensembles based on three performance indicators (F1, recall, and precision) evaluated for each class separately.
- Consideration of ensembles of neural networks (first level of fusion) based on weight matrices associated with performance metrics, networks, and classes.

Following the results, performance improvements were observed at each level. At the NN selection level, the three selected NNs had a penalty (sum of places) between 29 and 31, while the remaining six NNs had a penalty between 80 and 116, far from the first three. On the other hand, each selected NN contributes complementarily: ViT for capturing global relationships, DeepLabV3+ for contour refinement, and Attention U-Net for focusing on relevant areas. At the selected NN level, average performance values were obtained between 86.01 and 86.59 for F1, between 84.05 and 88.03 for recall, and between 85.26 and 88.17 for precision. At the first fusion level, average performance values were obtained between 90.41 and 90.50 for F1, between 89.79 and 90.08 for recall, and between 90.79 and 91.25 for precision. It is observed that the worst performances of the ensembles were better than the highest performances of the individual NNs. At the second level of fusion, the following average performance values were obtained for the global system: F1 = 93.53, Recall = 93.38, and Precision = 93.68. It is observed that the performance values of the global system were better than those of the ensembles in the first level and, obviously, better than the performance of the individual neural NNs. Compared to other results from similar works (Table 11), the performance obtained is good, considering that five classes were considered instead of two (as in other articles: tumor and non-tumor).

**Table 11:** Contextual comparison with representative AI-based studies in ovarian cancer imaging.

**Ref**.	**Classes**	Imaging	NN models	DB	Performances
Drury et al. (2025)	•High-grade serous ovarian carcinoma	Contrast-enhanced CT	3D U-Net	•OV04 dataset •Barts Health HS Trust	•*F*1 = 0.78 •*P* = 0.76 •*R* = 0.72
Kodipalli et al. (2023)	•Tumor •No Tumor	CT	•2D U-Net •3D U-Net	Owner	2D U-Net: •*F*1 = 0.918 •*P* = 0.938 •*R* = 0.946 3D U-Net: •*F*1 = 0.941 •*P* = 0.925 •*R* = 0.973
Hu et al. (2024)	•Tumor •No Tumor	Contrast-enhanced CT	Bayesian network	Owner	•*F*1 = 0.8631 •*R* = 0.8305
Liu et al. (2022)	•Tumor •No Tumor	CT	MGLU-Net	Owner	•*F*1 = 0.7614 •*P* = 0.8437 •*R* = 0.7208
Our	•No Tumor •Differentiated •Proliferative •Mesenchymal •Immunoreactive	CT	•Two fusion ensembles •Attention U-Net •DeepLabV3+ •ViT	TCGA OV	•*F*1 = 0.9353 •*P* = 0.9368 •*R* = 0.9338

To provide a broader context for the obtained results, Table 11 summarizes representative studies addressing ovarian cancer analysis using artificial intelligence and medical imaging. However, the reported performance values should not be interpreted as a direct benchmark or numerical ranking between methods. The included studies differ substantially in terms of datasets, patient populations, imaging protocols, target definitions, model architectures, and evaluation procedures. In particular, some previous approaches considered binary tumor/non-tumor classification or segmentation, whereas the present study evaluates a five-class representation incorporating patient-level molecular subtype-derived labels. Therefore, the comparison is intended to provide methodological and performance context rather than to establish superiority of the proposed framework.

The reported performance values originate from the respective publications and were obtained using different datasets, target definitions, imaging protocols, model architectures, and evaluation procedures. They are therefore presented for contextual purposes and should not be interpreted as a direct head-to-head comparison.

This study presents some methodological limitations, despite the encouraging results. It was done on a predefined dataset that is limited, and this can limit the applicability of the model to data from other sources or for other patients. For example, to improve the results, data (image) augmentation can be used. On the other hand, molecular subtypes were assigned through mapping derived from TCGA genomic classifications and not through direct imaging-based differentiation. Consequently, CT imaging alone cannot resolve transcriptomic heterogeneity within tumor regions. A methodological limitation of the proposed fusion strategy is that the same validation cohort was used both to select the three component networks and to derive the class-specific ensemble weights. Although the independent testing cohort remained completely isolated from these procedures, repeated use of a relatively small validation population may lead to optimistic model selection or weight estimation. The reported test results should therefore be interpreted in this context. Future studies should use nested patient-level cross-validation or a dedicated tuning cohort to separate architecture selection from ensemble-weight optimization. Probability calibration of the component networks should also be incorporated before probability-based weighted fusion, particularly when model confidence values are used directly in ensemble decision rules. Another limitation concerns the cross-study comparison. Because the previously published methods were not reimplemented and evaluated on the same patient-disjoint TCGA-OV split, the numerical results reported across studies are not directly comparable. Differences in datasets, patient populations, imaging protocols, target definitions, dimensionality of the models, and evaluation procedures may substantially influence the reported performance. Future benchmarking should therefore reimplement representative competing segmentation approaches under an identical experimental protocol. Future research integrating multimodal datasets, including histopathology and genomic sequencing, is required to validate subtype-specific imaging biomarkers and enhance biological interpretability.

## 6 Conclusions

The main contribution of the paper is the two-level decision fusion framework, developed to target the heterogeneity of ovarian tumors and the high interclass overlap present in CT images. It proposes a systematic selection of ensemble networks based on F1 score, recall, and precision for the five classes, considering an overall performance score. In this context, the proposed system aligns with the new trend of AI in radiology as a decision support. The results suggest that this ensemble technique, which combines multiple deep learning models, can improve performance in ovarian tumor segmentation. The use of patient-level molecular subtype information provides an additional label dimension for evaluating multi-class tumor segmentation, while its biological interpretation remains limited by the absence of direct voxel-level molecular annotation. As future work, multimodal integration can be achieved by collecting biological, morphopathological, clinical, and surgical data from the same patients, thereby contributing to diagnostic accuracy. The real-time operation of experimental models may constitute new research directions.

## Data Availability

The original contributions presented in the study are included in the article/supplementary material, further inquiries can be directed to the corresponding author.
